# Factoring ethics in management algorithms for municipal information-analytical systems

**DOI:** 10.1007/s43681-021-00098-5

**Published:** 2021-10-08

**Authors:** Kamolov Sergei, Kriebitz Alexander, Eliseeva Polina, Aleksandrov Nikita

**Affiliations:** 1 Public Governance Department, Moscow State Institute of International Relations, University of the Ministry for Foreign Affairs of Russia, 76 Vernadskogo Ave, Moscow, 119454 Russia; 2 Peter Löscher Chair of Business Ethics, Technical University of Munich, Arcisstr. 21, 80333 Munich, Germany

**Keywords:** Digital public governance, Intelligent systems, Information-analytical systems, Digital social media

## Abstract

The discourse on the ethics of artificial intelligence (AI) has generated a plethora of different conventions, principles and guidelines outlining an ethical perspective on the use and research of AI. However, when it comes to breaking down general implications to specific use cases, existent frameworks have been remaining vague. The following paper aims to fill this gap by examining the ethical implications of the use of information analytical systems through a management approach for filtering the content in social media and preventing information thrusts with negative consequences for human beings and public administration. The ethical dimensions of AI technologies are revealed through deduction of general challenges of digital governance to applied level management technics.

## Introduction: the relevance of artificial intelligence in public administration

Artificial intelligence (AI) is increasingly shaping how our societies and institutions are maintained, organized and managed. Hence, the consequences for the use of artificial intelligence bear major implications for public administration, in which AI is addressing greater numbers of individuals then in other cases [1]. In this context, the current COVID-19 crisis has demonstrated not only the urgency of ruling out smart technologies on the national level, but also illustrated the importance involving of local administrative units in the role out of AI in public administration [2].

Given the prominence of artificial intelligence in the public debate, one can observe an intensification of the debate on the principle-guided rollout of technologies, which are usually summarized as artificial intelligence. Consequently, the past few years have witnessed a surge in ethical guidelines for AI, including amongst many others the Asilomar AI Principles, the German Ethics Code for Automated and Connected Driving, Beijing AI Principles or the G20 Human-Centered AI principles. Some of the negative side effects of increased use of AI have been covered by this debate, including biases, data protection and the philosophical and ethical general limitations of outsourcing human tasks to machines and software solutions. As pointed out by earlier literature [3], these conventions and charts have concentrated on very general pillars of ethical regulation. Comparing the existing frameworks, Floridi et al. as well as Floridi and Cowls conclude that the ethical principles of AI can be subdivided in beneficence, non-maleficence, autonomy, justice and explicability [4]. Apart from the more general principles of AI, the debate on criticality of AI solutions has been rising. Criticality refers here to the likelihood that a particular AI solution is conflicting with the stated principles of AI governance [5]. However, some scholars have also pointed out that AI ethics failed not clarify its linkage to legal regulation and face many difficulties in terms of implementation [6].

The analysis of the relevant research base shows that AI ethics, especially in the area of public governance, is considered by most scholars based on particular cases of AI technology implementation. Given the availability of general theoretical recommendations on AI ethics in the scientific literature, there is no conceptual linkage between the AI ethics in general terms and the AI ethics for public administration. Taking into account this fact, we base our research on the consideration of a special case of AI ethics exercise—social media content filtering. The closer look for the sake of the present research is taken at the Russian case where public governance at the cities level show transparency in methodology and serve as a good reference point.

In our following paper, we tackle, therefore, the question how existing principles of AI ethics can be applied by public governance exemplifying the area of increased implementation of AI technologies in Russia—social media content filtering. To approach this question, our article will be structured as follows. First of all, we give a review of state-of-the-art literature in the field of information and communication technology (ICT) implementation in twenty-first century public governance, to show the need and relevance of the introduction of AI technologies in modern IAS. Then, we will shortly form our AI ethics framework and refer the principle of criticality, which has been used in some AI frameworks. In the second part, we discuss the implications of AI ethics in our case study of management algorithms for municipal information analytical systems in Russia. Here, we describe a management approach, which can help to tackle the ethical risks coming along with the increased use of ICT systems and emphasized the role of human oversight, when dealing with highly sensitive information.

## Information and communication technologies (ICT) in the context of public administration

In the field of ICT technologies development for public governance, there are obviously four main streams of scientific articles, and, accordingly, the authors' approaches to modern trends and the role of implementing AI technologies.

### The fundamental role of ICT

The generally accepted definition of AI is the ability of intelligent systems to perform tasks commonly associated with intelligent beings. However, the information and communication technologies (ICT) are worth considering due to the fact that they constitute the basis allowing the functioning of AI. Information and analytical systems, in turn, are a software package that allows real-time analysis of specific data with a given level of detail. IAS are currently considered as platforms for the introduction of AI technologies to expand their functionality. While the automated decision-making tools are considered as a so-called culmination in development of ICT, IAS and their integration with AI. Automated decision-making tools are autonomous self-learning systems that gather and process data to make qualitative judgements with little or no human intervention [7]. Focusing on governance, in particular public governance in the twenty-first century, it is impossible to avoid mentioning ICT. The ICT infrastructure fully characterizes modern “digital” public governance. The necessity, and even the inevitability of ICT implementation is reflected in the articles of many modern authors such as Dahiya and Mathew [8]. The research conducted by Verdegem and Verleye develops the concept of ICT in public administration, focusing on the use of technological solutions to improve the quality of public services, engage citizens, and define an end-user-citizen approach to governance [9].

The emergence of the “smart city” phenomenon, and hence “smart governance”, has brought the role of ICT infrastructure to a new level. Public policy within the framework of smart governance, according to Pan and Wu, should be aimed at ensuring benefits for citizens, based on citizen needs’ predictive analytics technologies as for modelling of their requests [10]. In addition, researchers as Cortés-Cediel, Cantador and Gil hold an opinion that the scenarios of interaction between the state and citizens will inevitably become more complicated and should be provided with modern technologies [11].

### The role of social media as a tool for personalized governance

The current trend towards individualization and personalization of governance, focusing on citizens, requires up-to-date data on the needs of citizens. Such data can be obtained in particular through the analysis of profiles in social networks and the media space, as the main source of “direct communication” with consumers of services.

In particular, Zavattaro and Brainard believe that the analysis of social networks, especially user preferences, serves as a necessary basis for the formation of both a general idea of the needs and the individualization of the process of providing public services [12]. The technological basis of this approach, according to Logesh et al. in most cases is presented by a recommendation system [13]. In this particular case, the authors use the travel system to sample recommendations for visiting places of interest based on social network data and geo-position.

### Platform-oriented approach to government technologies

Platforms in public governance represent a single digital field for interaction and mutual enrichment of data. In public governance, platforms are used primarily to improve the efficiency of working with citizens and businesses, and, therefore, the entire governance process.

Styrin, Dmitrieva and Sinyartullina argue that nowadays government digital platforms can become an innovation disruption, transforming the relations of government bodies, business and society, primarily in terms of reducing the costs of interaction, increasing transparency and public control over government decisions and the provision of services [14]. According to their view, government digital platforms play a crucial role in terms of public services delivery tailored to the needs of citizens. Al-Hassan, Lu H. and Lu J. discussing the conceptual structure of e-government and the personalization of public services, define government digital platforms as the technological basis for collecting relevant data, operational interaction and responding to the needs of citizens and business [15]. Developing a multilayer digital platform for smart city management, Chamoso et al. highlight the need to personalize public services, proactively provide them, and introduce predictive analytics to define the needs of citizens and businesses in advance [16].

### The role of AI in modern governance, business and social development context

Recent research in this field argue that AI is an innovative technology with a high potential for improving the efficiency and productivity of management not only for public governance and corporate management, but also for the development of the society. There is also a trend theory that the pace and direction of AI development will determine the pace of development of the entire society in the near future.

In particular, Mikalef et al. define universal criteria for development of AI capability through the following categories of resources: tangible resources (data, technology, and basic resources), human resources (business and technical skills) and intangible resources (inter-departmental coordination, organizational change capacity, risk proclivity) [17]. Duan et al. in turn, investigate the role of AI in the era of big data. In particular, a critical analysis of AI in the field of decision-making technologies is given, as a result of which the authors argue about the irreversibility of the development of such technologies and the replacement of a number of human functions, in which the decisive role should still remain with a person, and AI should be considered as a function rather than an end in itself [18]. Dwivedi et al. provide a general conceptual analysis in the modern context of social development. Their vision is focused on AI as a high potential technology that should be used as a basis for future innovative development [19]. However, the authors also pay attention to the importance of human control, awareness of risks and the development of a strategy for managing them.

Considering that the majority of researchers address the introduction of AI both in the public sector and the private sector mainly in terms of specific cases, it is crucial to note a number of articles that provide a comprehensive description and understanding of the importance of relevant officials’ request for the use of such technologies [20]. The idea is to consider AI with its complex effects and the difficulties that arise in implementation. Thus, a model of influencing factors for the adoption of AI in municipalities developed and extended by Schaefer et al. provides a framework of perceived challenges employees are facing when adopting AI through the prism of aggregated dimensions [21]. The proposed model offers an orientation guide for municipalities that are switching to AI technology. Expanding the methodology, Mikalef et al. investigate the competing enablers and inhibitors that influence AI capability levels in municipalities [22]. Particular emphasis is put on such factors as perceptions of value and organizational innovativeness, impact of top-government decisions on actions of lower level administration, perceptions of citizen and governmental pressure.

On the other side, practical research [23–25] demonstrates that the topic of AI ethics public governance is addressed by most modern scientists through the prism of specific areas of AI application. This gives grounds to conclude that a unified approach to AI ethics in modern public governance have not been established yet. In this regard, the social media serve as one of the most spread and significant areas of AI application [26], since in the modern world social networks are not only a means of ensuring the connectivity of society, but also an instrument of state policy, personalization of public governance, detection of offenses, as well as ensuring the open governance. In this regard, a number of researchers address the issue of AI responsibility. Currently, the issue has not yet been resolved. Despite the benefits AI provides in terms of data processing and decision-making support, the matter of responsibility still remains, since the mass introduction of AI has already begun in key areas, such as healthcare, where defects are unacceptable and human control of AI is so far inevitable [27]. Tigard, speculating on the principle of AI responsibility, argues that it is a complex concept that should be considered from several angles: normative, possessive and descriptive responsibility [28].

Despite the variability of approaches, all of them are united under a leading trend—personalization of governance activities to form an individual approach to each object. To achieve there is a need of up-to-date data and decision support technologies on technical level. The obvious solution for such a request is the AI technology, including machine learning, working with big data, predictive analytics. Such technologies are increasingly used or planned to be used in the framework of governance activities, but still present difficulties in terms of implementation, standardization of solutions and approaches to regulatory policy of this area. To address these issues, first of all, there’s a need to understand and agree on a unified approach to the exploitation of AI technologies at least in public governance.

## Factoring AI ethics with public administration

So far, specific ethical frameworks concerning ICT technologies have not been established in literature. We, therefore, orient ourselves to the existent literature on AI ethics, as it concerns the more general case. As a starting ground for AI ethics, we chose the AI4People framework “AI4People—an ethical framework for a good AI society: opportunities, risks, principles, and recommendations” [29], which has established general principles for AI management. We opted for this particular framework, given the fact that it is itself based on the comparison of different AI frameworks. Moreover, earlier research [30, 31] has used the framework to grasp the different implications of AI governance. As mentioned, the AI4People consists of the pillars beneficence, non-maleficence, autonomy, justice and explicability. These five principles require a short explanation, especially in the context of public administration.

*Beneficence*: the idea that AI should benefit individuals is widely shared acknowledged by all major parties and central part of most AI for Good approaches. The specific understanding of goodness is often linked to pre-existing concepts such as the realization of the United Nations Sustainable Development Goals or the fulfilment of constitutional and human rights [32]. In this sense, the development of AI is integrated in the discourse on development and human rights, explicitly in their interpretation as socio-economic or access rights such as right to health, right to development or right to life [4]. This bears major implications for public administration, which has to demonstrate that using AI constitutes an improvement to traditional ways of public governance and that it conforms with the constitutional rights of the citizens. Moreover, public administration has to contribute to the realization of normative goals such as enhanced public participation [33] or socio-economic improvement.

*Non-maleficence*: the principle of non-maleficence implies that artificial intelligence should not be directed against human beings or their rights [34]. This could include for example the right to self-expression and free opinion. In its application, the non-maleficence principle differs fundamentally from the beneficence principle, as it formulates lines that AI development and research should not cross [31]. For public administration, the notion of non-maleficence is noteworthy in the context of defence rights, which limit state interference. The harm for life, liberty and property, which could arise from AI has, therefore, received considerable attention from the AI ethics discourse.

*Autonomy*: autonomy traditionally refers to the capacity of an individual to make an “informed, uncoerced decision” [35, 36]. Floridi et al. argue that “affirming the principle of autonomy in the context of AI means striking a balance between the decision-making power we retain for ourselves and that which we delegate to artificial agents” [4]. In this sense, the principle of autonomy is a principle, which aims to mitigate the risks of AI use cases, which render individuals to tools, or to create the sentiment that human beings are exposed to AI solutions. This is highly relevant for state action, which is based on the idea of government by consent and which has—given the power asymmetry between state and citizen—to emphasize on autonomy more than commercial AI solutions, where individuals can choose whether the accept the terms of conduct or not.

*Justice*: as it is the case with the principle of autonomy, the justice principle is more difficult to grasp than beneficence or non-maleficence, given the fact that there is no single definition of justice, which reflects all of its aspects. In the debate on public administration, the justice criterion refers to the distribution of services and goods to the citizens and to customers. In specific, providers and users of AI solutions are obliged to avoid discrimination and to reduce potential sources of bias [37]. The crucial aspect is here is the equality of access: AI should treat all individuals in the same way. Discrimination by AI solutions has many different causes and may have gender bias implications or affect people of racial, ethnic, national or social origin, such as communities of Asian or African origin, Roma, indigenous peoples, Aborigines and people belonging to different castes [38]. The recent discourse in the United States on the issue of race, but also existing cases of unfair treatment due to the wrong calibration of data demonstrate here the relevance for embedding the justice principle in the use of AI in public administration [39].

*Explainability*: the principle of explicability, or explainability has been established in the conceptual paper of Floridi et al. with the purpose of “enabling the other principles through intelligibility and accountability.” The importance of this relates to the black box character of AI. Even before the advent of AI technologies, certain products were required to meet specific standards on transparency and explainability [40]. This applies for example to pharmaceutical products. In essence, this means that decisions by AI need to be understood by decision-makers and users of AI solution. Transparency is in specific demanded in the area of public governance, deriving from the rule of law and rule by law principles [41].

Given the considerable room for interpretations, which is left by these more general principles, decision makers require an overview on how to tackle the different use cases of AI and to find out in which degree the AI use case is colliding with the stated AI principles [42].

*Criticality*: the notion of criticality refers here to the likelihood that a particular AI solution is conflicting with the stated principles of AI governance. The notion of criticality appears in the frameworks of Smart Dubai, Singapore AI frameworks and the Opinion of the German Data Ethics commission. According to the German Data Ethics Commission, “system criticality is determined by assessing an algorithmic system’s potential for harm, on the basis of a two-pronged investigation into the likelihood that harm will occur and the severity of that harm” [43]. Hence, system criticality generates major implications for the measures, which need to be taken into account to mitigate risks posed by artificial intelligence. The Singaporean framework for example distinguishes between the severity and the probability of harm (see Table 1):

**Table 1:**
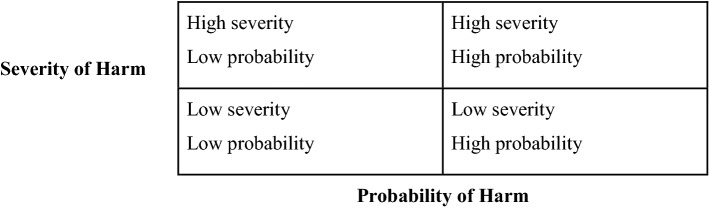
Singapore framework—user case distinction

Nevertheless, the clear meaning of system criticality or the distinction of different use cases necessitates further elaboration. Moreover, the implications of criticality are also relevant in terms of the classification within different use cases of one AI solution and public administration in specific, given the asymmetry of power between citizen and state.

## Model: the use of ICT technologies in the Russian context

To present an apt example for gauging the implications of AI ethics for public governance, we focus on the increasing use of AI by the public sector and by state-owned companies in Russia [44]. One important aspect concerns here the evaluation of data from social networks to identify the nature of news feeds by information and analytical systems (IAS). The Russian context might contribute to the general debate on ICT technologies for two reasons: first, Russia’s recent AI strategy has paved the way for rolling-out of AI technologies and smart city solutions on the local level, which is why local decision-makers have to implement management approaches for using these technologies. Local self-government is guaranteed by the Russian constitution [45], what concerns also the communication of Russia’s 20,846 municipalities to their citizens [46]. Second, Russia has a very specific social network culture, given the multitude of domestic social networks (e.g. Vk.com, Odnoklassniki.ru and Yandex. Regions) and communication applications (e.g. Telegram and Viber). The following model serves therefore as a proposal for how to manage risks related to the implementation of IAS systems and might be used for other cases as well. The model presented here lines out the purpose of IAS technologies, the way to categorize between different types of news feeds as well as evaluation their criticality and procedures, which need to be established to guarantee human oversight and operational efficiency.

### Understanding the purpose of information and analytical systems in public administration

When implementing ICT technologies in specific, it is important to understand the implications of ICT technologies on the local level and the potential effects these technologies [47] might have on the norms of AI ethics. In general, the information environment of municipalities embraces a complex of interconnected multi-functional systems: information and analytical systems (IAS), monitoring, information processing systems, communication and social networks. Those elements can be in public, municipal and private ownership. Information and analytical systems are designed to promptly formulate, correct and monitor the implementation of tasks to respond to positive and negative news feeds, as well as to facilitate or automatize setting, adjustment and control over the implementation of such tasks.

We refer to a news feed as a significant event, phenomenon that has occurred, a fact of social life or personal experience that became the reason for publication on the net and is reflected in the information background of the municipality. Depending on the situation, news feeds can be positive and negative (an incident). The latter usually requires responsive actions on behalf of the municipality to communicate actions or guidance.

The IAS should be deployed in accordance with the omni-channel principle, that is, it should be technically able to receive signals (messages) from citizens in almost any convenient way (via any available communication channels including social networks).

Special monitoring systems (SCAN, Brand Analytics, Medialogia, TGStat) collect and provide real-time analysis of public information from the social media. For instance, TGStat covers and maintain open-access statistics of over 70,000 Russian-language Telegram channels. Social media are primary sources of news feeds for any regional or local governments and include social networks, forums, chats, microblogs, video hosting, Telegram channels, news agencies and thematic portals. These sources should be considered as both information inflow channels and broadcast means to publish open information by local authorities and municipal organizations. Integration of the official pages into social media context is becoming an important issue for public governance.

Some information sources can be referred as toxic sources or authors, when they pursue the goal of propaganda, manipulation, and imposition of their subjective and biased point of view. One contemporary example is the use of online propaganda by terrorist organizations, which aim to disseminate propaganda to the public and to increase their media presence in the society [48]. Moreover, the debate on social media and fake news has surfaced in the context of the American elections in 2016, what includes the dimension of cyber stability and an emerging non-traditional understanding of political stability [49].

The potential harm for individuals and the society, which could originate from malicious newsfeeds, explains therefore the need of technological solutions, which distinguish between different types of news feeds according to their potential harm. The degree to which a software solution and a respective management approach are realizing these goals, is reflected in the principle of beneficence, as it satisfies the goal of transparent administration and prevention of damage.

### Categorizing news feeds

The potential threat, which could emerge from harmful agents, necessitates the information and analytical systems (IAS), which are able to categorize between different types of news feeds. Literature has established different division lines of distinguishing between different ways and kinds of communication [50] and communication styles [51]. Fundamentally, we elaborated three categories of news: factual, emotionally coloured and information thrusts. Given the lack of literature on the categorization of news feeds in IAS, but the broader distinction between factual and emotional communication styles [50, 51], the elaboration is derived from the analysis of possible algorithms and actions that any public governance system would take. The closer look for the sake of the present research was taken at the Russian case where public governance at the cities level show transparency in methodology and serve as a good reference point.

A factual news feed (FNF) is an event in the Internet that does not have a pre-shaped emotional colouring and carries an informational character (statistical news bulletins, weather data, published reports) [51–53]. The purpose of the FNF is to inform the audience, citizens. However, when reading, it can evoke a certain perception or response. Therefore, FNF can be divided into three groups: creating a positive information field, creating a negative information field and mixed FNF.

Most often, conditionally positive FNF do not relate to the socio-economic and political spheres, but rather to technology, science, stories of mutual assistance and feelings for solidarity. In the vast majority of cases, such FNF do not require a response [54] and can serve as the basis for a positive news agenda for the municipal district.

Negative factual news (factual incidents) most often relate to socio-economic and political spheres, or ecology. This type of FNFs can be classified into the following sub-categories:

– Emergency is an extremely important and significant FNF that points to the life and health highest threats, massive human rights abuse, political stability or a high risk of an economic crisis. The reaction strategy will involve official statements by press services and/or officials, inspections, departmental and financial engagement;

– Security is an important and weighty FNF, which has to do with signals of threats to the life and health of a large group of the population, and basic human rights violations. The response strategy includes official statements from press services, solution responses, analysis and responses to negative messages, verification and subsequent confirmation or disavowal of problems, departmental and financial engagement [55];

– Comfort an event that is often associated with housing and communal services, transport matters. The response strategy focuses on monitoring and “problem-solving” when needed, including responding to negative comments;

– Current matters rarely cause an emotional response from a large number of citizens, require monitoring and address in case of need of negative narrative.

Mixed FNFs do not evoke an emotional response directly by their content; however, they often attract a response from readers by the presence of a specific personality or organization in it. The preferred online response strategy would be monitoring and reaction when needed and gaining weight, as well as working out negativity when needed.

*Emotionally coloured news feed* (ENF). Emotions have been widely discussed in the context of social media [50, 51, 56, 57]. The author of an emotionally coloured news feed seeks to convey his/her attitude and point of view to the reader, often shaping subjective a priori vision of the situation. ENFs can be of two types: positive and negative. Positive ENFs are not frequent and will mainly represent gratitude. The strategy of municipal officials, in this case, would be the recognition response. Negative ENFs are grouped into claims, complaints, and requests. Claims are characterized by high emotionality and publicity (comments, posts on personal pages, “open letters”). They are aimed at the municipal employees or organizations who performed the work and do not contain constructive comments. An appropriate strategy is monitoring and dealing with the negative ones, as well as the implementation of departmental engagement offline. Complaints are directed at the very fact of problems, not at the employees [48]. It is possible to subdivide complaints into justified (associated with an unplanned or unknown fact that happened objectively) and expected (associated with a planned or already known fact). A response strategy in both cases would be an acceptance or resolution and an offline departmental engagement [58]. Requests with sufficient information literacy are personal messages on the official information interfaces. The required strategy would be answer-resolution or answer-acknowledge actions.

*Information thrusts* (ITh) are here defined as artificially created and pushed news feeds aimed at promoting false information, propaganda, panic creation, involvement in protest and/or terrorist movements, reputation and information attacks. Thrusts are used to significantly change the reputation of individuals, enterprises and organizations, public and municipal authorities, or generally create a negative information background [59]. In this sense, they cannot be fully grasped within the already existent continuum of factual and emotional communication [50, 51]. These actions can fall under legal norms of the penal code and might bear legal consequences. Thrusts can be classified as follows:

– Attacks on specific individuals (heads of enterprises, state or municipal employees, media persons). Causes: unsuccessful statements, immoral behaviour, acute injustice;

– Attacks on enterprises and authorities (city or district administrations, corporations). Causes: failures, errors, corruption, sanctions;

– Long campaigns a series of thrusts, selection of negative topics that cause a "viral" growth, buzzing the topic for months.

Monitoring would be an acceptable strategy in the case of a small-scale attacks, and in case of growth (reposts with exponential growth), an official refutation answer may be needed.

### Building up the news feed processing cycle

The IAS solution alone is not sufficient, when it comes to determining the emergence of a news, which is why the treatment of news feeds and its analysis requires a special management approach by public administration, to mitigate conflicts with AI principles. Human oversight plays, therefore, an important role to guarantee that the IAS fulfils its purpose and to prevent infringements in the rights of other parties. Based on this, we derive that news feed processing therefore requires seven successive stages creating a consecutive cycle:

1. The emergence of a news or an incident and its recognition by the monitoring system;
2. Definition of the news feed category;
3. Determination of the critical level of the news feed;
4. Choosing a response strategy;
5. Reaction/implementation;
6. Control and follow-up analytics;
7. Corrective internal management actions.

The primary processing of the received signals, as a rule, are outsourced to third parties, on the basis of Service-level agreement (SLA)—agreements on the required level of information processing with specified parameters of the quality of the services provided. It regulates the subjects of provision, the terms and scope of processed information as well as methodology for measuring the level of quality of services, methods of quality control, guaranteed availability of the contractor, responsibility of the parties. In essence, the organization involved on the basis of the SLA plays the role of an on-call desk, or a support service (Help Desk)—the first line of response, responsible for an immediate recognition, reconciliation of duplicate news feeds, their departmental and territorial binding, dispatching, incoming messages routing.

The style of communication (tone of voice) when addressing the incoming information plays significant role to reach citizens consent related to the local governments action. This is important in the context of explainability debate, which implies transparency on the use digital technologies by local authorities. Consequently, the tone of voice of municipality and its organizations is based on three elements: clarity, benevolence and efficiency. Specifics of the tone of voice are reflected in the Table 2.

**Table 2:** Communication styles specifics

Elements	Expected communication style	Communication style to avoid
Clarity	• Simple syllable • Clear indication of the sequence of the process (for example, for the improvement): the customer, what will be done and in what time frame • Clear definition of departments and services engaged in the resolution of the incident	• Official and bureaucratic • Vague wording about the temporality of inconveniences • Empty messages promising the improvement
Benevolence	• Empathy for the applicant/caller • Attentiveness and compassion • Address to the applicant by name, gratitude for active participation in the life of the district, city, municipality; apologies for a specific situation • Use of predicates to emphasize the actions taken by the authorities: “redone”, “fixed”, “created an application with a number” • After resolving the incident, provide the necessary contacts for the future	• Passive aggression • Ignoring appeals/incoming messages • Lack of personal approach, of apologies for the inconvenience • Anonymity of feedback • Silent resolution of the problem
Efficiency	• Informing the applicant about recognition of the matter, for the transparency of the process, indication of the responsible organization/official • Proactive approach to meet deadlines: the day after the promised fixing period, be the first to write what the delay is about • Informing citizen/applicants about the performance of the promised work, preferably with a photo report • Cooperation with the media according to the critical level of the incident	• Acceptance for work without informing the citizen • Reactive approach to meeting deadlines • Silence of success. Unannounced problem resolution • Protecting employees in any situation or ignoring problems with employees • No explanations, no recognition of the responsibility

Once news feed processing cycle reaches accomplishment phase, local authorities and municipal organizations should initiate monitoring of the media for further publications, introduce clear management adjustment procedures where appropriate and establish follow-up communication channels. Valuable information can be retrieved from reference frequency in the media, its tonality, and citations of top officials. Three core analytical indicators are presented in Table 3.

**Table 3:** Core analytical indicators of post-news feeds

Indicator	Purpose
Sentiments	To analyse the dynamics of positive and negative news stories coverage
Dissemination and reactions	To analyse audience reach, number of “likes”, “reposts” and comments on social networks and media resources
News feeds by area	To analyse the number of news feeds in the relevant areas

Moreover, local authorities might opt to disclose the functioning of IAS software solutions, to enhance transparency towards the citizen.

## Discussion

Based on the preceding elaboration on news feeds and their classification, we discuss how to realize the notion of criticality, which has been set forth in AI ethics, in the context of IAS.

### Determining the critical level of the news feed

An analysis of the news reference quality adjusted by the sources—to prevent biases—allows assessing the relative importance of a news and its potential implications for the society. The main issue could be here that automated systems have a bias, when classifying the data, leading to false alerts. The general area of communication in social media here is highly important, as it is closely related to the right of self-expression and opinion [60]. At the same time, certain types of news feeds can bear negative consequences for the society, such as propaganda launched by terrorist organizations or news feeds conflicting with the personality rights of individuals [61]. Balancing between the different implications requires, therefore, a strategy that distinguishes between different levels of harm, which are created by a news feed, and respective measures to be taken in response. This is not only important for maintaining the principle of fairness [31]—understood as the wrong classification of a harmless newsfeed as dangerous -, but also for realizing the criterion of beneficence [29], the notion that AI has to provide an added value such as filtering out potentially harmful news feeds.

For bridging both potentially conflicting mission statements of IAS, criticality defines the required initial reaction time of the municipal authorities to avert damage from others. The shorter the required initial reaction time, the more critical is a specific situation. A further criterion for specifying the criticality of a situation is “conflict intensity”. This links up to the understanding of criticality in the AI discourse and the scheme of the Singapore AI Governance Framework that has connected criticality to “harm” [compare: 66; 3.15]. In the following, criticality or severity are not only understood as principle limiting the use of AI and for determining of the degree of human involvement, but also for determining the urgency of responding to a potentially harmful news feed. The exposure of individuals to propaganda material of terror organizations or other information thrusts conflicting with personality rights might amount to irreversible harm to an individual. Based on this, we derive four critical levels of the news feed (Table 4). The critical levels are based on the idea that IAS should only intervene when it is necessary and to avert damage.

**Table 4:** News feed critical levels

Critical level	Criteria	Engagement recommendations	Expected reaction time
Level 4	Low critical level with narrow audience coverage, slow spread of information in the media, lack of publications from “toxic authors” and “independent” sources, no disasters with victims, coverage of residents in concern less than 10 thousand	Direct communication with citizens at the level of municipal organizations and in the community sites. Prompt response to the news feed should be disseminated through information channels interested in covering the event	4 h
Level 3	Average critical level with increasing reach to the audience/increasing spread, anticipated publications from “toxic authors”, coverage less than 50 thousand	Need in inspector and representatives visits from the responsible organizations to the scene of the event. Publication of news by respective organizations with feedback on the resolution of the situation. If the incident was not resolved in a positive manner, then the problem must be addressed at a higher administrative level	2–3 h
Level 2	High critical level. A wide audience reach, high spread pace, publications from “toxic authors” on “independent” sources, highly politicized. Coverage of citizens above 50 thousand	Prompt response. When addressing, to be guided by the national emergencies protocols. Need to establish effective communication with representatives of the media and the public	1 h
Level 1	Maximum critical level, a wide audience reach, high spread of information, publications from “toxic authors”, highly politicized or refers to disasters with victims, coverage of citizens over 100 thousand	Highest attentiveness. Focus on productive communications with the media and the public concerned in view of the resonance of the topic	0.5 h

### Typology of strategies for news feeds processing

For mitigating potentially arising conflicts between the notion of freedom of opinion and the need of public administration to prevent certain information entering the society, we propose six possible strategies for responding to news feeds: events monitoring, acknowledging the problem, response containing a solution of the problem encountered, refutation, communicating positive news, active counteraction to information attacks.

Monitoring is the systematic observation of the state of local affairs and track of qualitative and quantitative changes in the media field (messages, mentions, publications, events). Monitoring is becoming an automated, in some cases AI, real-time function. When processing messages, it is important to carry out classification discussed above and to assess the influence of the media (the presence of “direct speech”—statements of eyewitnesses, interviews with witnesses of events). The monitoring strategy is sufficient for positive and mixed news feeds. There are four monitoring phases: observation of potential sources of events, accumulation of material, filtering and categorization, transfer of the collected material for further analysis.

Acknowledgment of issues, or recognition response is an umbrella term that combines types of reactive feedback that do not require emergency corrective action, due to existing plans or actions of improvement in the relevant areas. The acknowledgment response is used in case of pending complaints or positively coloured FNFs and is carried out by subordinate organizations. The key result of the recognition response is the communication of basic information and improvement of the image of public servants and employees of subordinate organizations. Expected publication time of such responses: 4 h.

### Acknowledgment response template

Resolution response is a term for types of active feedback to the citizens that require offline corrective actions, taken at the level of subordinate municipal organizations. The key performance indicator is a timely response and settlement of current problems. The expected resolution time is less than two hours with the news broadcast for citizens within one or two working days. After ensuring the immediate response, the issue should be submitted for a follow-up by subordinated bodies, informing the citizens about the actions taken. An expert assessment of what happened might be necessary if a news feed attracts high social attention.

Refutation strategy is applied in the case of large-scale information attacks. The refutation is carried out by involving a third-party independent expert and/or presentation of factual (documentary) materials, indicating an attempt by identified or unidentified persons to mislead citizens and spread false information in the net.

Communication of positive news is carried out by subordinate municipal organizations through press events, social networks and messengers.

Strategy: active counteraction to information attacks.

Counteraction to information attacks implies preparation and distribution of official comments responding negative publications and/or publication of refutation materials. This strategy is advisable if:

- News feed related to the activities of local government or subordinate municipal organizations has become or can potentially become viral (i.e. articles, images or videos are distributed at high speed using social media algorithms or directly from news feed sources);
- In publications or comments related to the activities of municipal organizations, local top officials are mentioned in a negative way in the context of the efficiency of the city economy;
- Publications on the official pages of municipal organizations, citizens communities unfold an emotional negative discussion (more than ten comments within 3–5 h from the moment the news was published);
- Topic of the publication is under special control (seasonal issues, replicated themes and discussions).

Thus, the analysis of the existing practice of AI application through IAS in the field of news feed processing was carried out. The conducted case study presents a scientific value in terms of bridging the ongoing discourse between AI ethics and public administration in the sphere characterized by significant level of AI dissemination which is of particular value for public governance for ensuring security and rational development of society. Our conclusions and proposals will help AI tech providers to better understand the requirements of public governance and offer better thought-through systems to local and regional governments. Governments can also pilot a managerial approach to the functional scope of AI application at the practical level of public governance.

## Conclusion

Based on the preceding elaboration on news feeds and their classification, we discussed how to realize the principle of criticality set forth in AI ethics in the context of IAS. As described, the notion of criticality has been intensively discussed in context of the Singapore AI framework and the German Opinion of the Data Ethics Commission, which have been both discussed in AI ethics literature and belong to authoritative normative positions in the global AI discourse [62, 63]. Moreover, both codifications entail deep insights in the description and explanation of the terms “criticality” or “severity” [58, 64].

The purpose of using IAS systems is to filter the content in social media. Here, we distinguish between factual news feeds, emotionally coloured news feeds and information thrusts. The main ethical aim of filtering content in social media is to prevent information thrusts with a negative impact on the safety and security of the society. Social media can here be instrumentalized, to create panic or to distribute information with malicious intent such as propaganda of terror organizations. Realizing this aim requires a management approach, which distinguishes between different levels of “urgency” and “conflict intensity” and corresponding strategies, on how to engage with incoming news feeds. Automated decision-making tools might reach here some boundaries. To prevent biases and to maintain control over the system, human oversight, which is embedded in the strategies, plays a crucial role. Consequently, the necessity of human oversight has major implications for the training of employees in public administration, who have to be knowledgeable about the constitutional purpose of IAS systems and their potentially negative effect on human rights, but also of the existence of potential biases in systems filtering news feeds.
